# A Comparative Analysis of ChatGPT and Medical Faculty Graduates in Medical Specialization Exams: Uncovering the Potential of Artificial Intelligence in Medical Education

**DOI:** 10.7759/cureus.66517

**Published:** 2024-08-09

**Authors:** Gülcan Gencer, Kerem Gencer

**Affiliations:** 1 Department of Biostatistics and Medical Informatics, Faculty of Medicine, Afyonkarahisar Health Sciences University, Afyonkarahisar, TUR; 2 Department of Computer Engineering, Faculty of Engineering, Afyon Kocatepe University, Afyonkarahisar, TUR

**Keywords:** chatgpt, innovation, lifelong learning, learning opportunities, qualified teachers

## Abstract

Background

This study aims to evaluate the performance of ChatGPT in the medical specialization exam (MSE) that medical graduates take when choosing their postgraduate specialization and to reveal how artificial intelligence-supported education can increase the quality and academic success of medical education. The research aims to explore the potential applications and advantages of artificial intelligence in medical education and examine ways in which this technology can contribute to student learning and exam preparation.

Methodology

A total of 240 MSE questions were posed to ChatGPT, 120 of which were basic medical sciences questions and 120 were clinical medical sciences questions. A total of 18,481 people participated in the exam. The performance of medical school graduates was compared with ChatGPT-3.5 in terms of answering these questions correctly. The average score for ChatGPT-3.5 was calculated by averaging the minimum and maximum scores. Calculations were done using the R.4.0.2 environment.

Results

The general average score of graduates was a minimum of 7.51 in basic sciences and a maximum of 81.46, while in clinical sciences, the average was a minimum of 12.51 and a maximum of 80.78. ChatGPT, on the other hand, had an average of at least 60.00 in basic sciences and a maximum of 72.00, with an average of at least 66.25 and a maximum of 77.00 in clinical sciences. The rate of correct answers in basic medical sciences for graduates was 43.03%, while for ChatGPT was 60.00%. In clinical medical sciences, the rate of correct answers for graduates was 53.29%, while for ChatGPT was 64.16%. ChatGPT performed best with a 91.66% correct answer rate in Obstetrics and Gynecology and an 86.36% correct answer rate in Medical Microbiology. The least successful area for ChatGPT was Anatomy, with a 28.00% correct answer rate, a subfield of basic medical sciences. Graduates outperformed ChatGPT in the Anatomy and Physiology subfields. Significant differences were found in all comparisons between ChatGPT and graduates.

Conclusions

This study shows that artificial intelligence models such as ChatGPT can provide significant advantages to graduates, as they score higher than medical school graduates. In terms of these benefits, recommended applications include interactive support, private lessons, learning material production, personalized learning plans, self-assessment, motivation boosting, and 24/7 access, among a variety of benefits. As a result, artificial intelligence-supported education can play an important role in improving the quality of medical education and increasing student success.

## Introduction

Large language models (LLMs) such as ChatGPT represent a significant revolution in the field of artificial intelligence and are effectively used in text-based tasks. These models are deep learning-based artificial intelligence systems that learn from vast amounts of text data and can be employed to analyze, understand, respond to, or classify text-based problems. Models such as ChatGPT have been successfully utilized in various applications, including text generation, question-answering systems, translation, and language understanding. Additionally, LLMs have potential applications in many industries, including law, medicine, finance, education, and more. LLMs represent a significant advancement in the field of artificial intelligence. LLMs are artificial intelligence models that learn from extensive text data and can generate, respond to, or classify text in a human-like manner [1,2].

LLMs are capable of processing large text datasets as they are trained on millions or even billions of text examples. They learn the structure and semantics of language, making them capable of working in multiple languages. LLMs can also be fine-tuned for specific tasks, providing adaptability for particular applications [2,3].

One example of an LLM is the Generative Pre-trained Transformer 3 (GPT-3). GPT-3, developed by OpenAI, is a prominent LLM that has been used in various applications. It can generate response texts, articles, and even written content across numerous domains. Bidirectional Encoder Representations from Transformers (BERT), developed by Google, is an effective LLM used in tasks such as text classification, semantic analysis, and language understanding. Text-to-Text Transfer Transformer (T5), developed by Google Research, is designed for understanding text and performing text-to-text translation tasks. LLMs can also find applications in the field of law. They can assist in tasks such as analyzing legal texts, translating legal documents, and answering legal-related questions. LLMs can be utilized to aid students in legal education and practice. They are versatile artificial intelligence tools that can be highly effective in text-based tasks and hold great potential in various industries, including law [4-6].

Artificial intelligence technologies, as a constantly developing field, have made significant progress in education, health, industry, and many other sectors. These technologies, especially in the field of education and learning, have great potential to provide students with personalized learning experiences, optimize learning processes, and improve the quality of education [7]. This study aims to investigate how artificial intelligence technologies, especially artificial intelligence tools such as the ChatGPT model, can contribute to medical education and examinations. Considering that the academic success of ChatGPT has already been proven in a wide range of areas, this study examines the performance of ChatGPT in the medical specialty distribution exam that medical school graduates take to determine their areas of specialization.

Medical education is vital to human health and care, and various examinations are required to evaluate the knowledge and skills of medical students. In this context, the main objectives of our study were determined as follows: Measuring ChatGPT’s level of knowledge in medical sciences: (1) To evaluate the level of knowledge of this artificial intelligence model in the field of medicine by analyzing ChatGPT’s answers to various questions in the fields of basic and clinical medical sciences. (2) Comparing performance: To reveal the strengths and aspects of artificial intelligence that need improvement by determining in which areas ChatGPT performs better or weaker compared to medical school graduates. (3) Exploring potential applications of artificial intelligence technologies: Examining the potential applications and advantages of artificial intelligence tools in medical education, and exploring how integrating these technologies into medical education can contribute to student learning and exam preparation.

Hence, this study aims to provide valuable information on how artificial intelligence can be integrated into medical education, contribute to educational processes, and highlight innovative applications in this field.

## Materials and methods

Study approval was received from the Afyon Kocatepe University Scientific Research and Publication Ethics Board (approval number: 2024/13).

The medical specialty examination (MSE) in the field of medicine is an academically challenging assessment that medical school graduates can take at their discretion. As the name suggests, the MSE is a test that medical students take to gain specialization in various medical fields. This examination was introduced to address the increasing number of medical school graduates and the limited availability of specialist positions. The first examination in this field was administered in 1987. Candidates who achieve a satisfactory score in the examination are eligible to serve in training and research hospitals, medical faculties, or the Institute of Forensic Medicine, all under the jurisdiction of the Ministry of Health. Following this, they obtain the title of Specialist Doctor in the field they choose. The sole requirement to select a medical specialty and receive specialist training is to pass the MSE. The examination encompasses a wide range of subjects, both in clinical and basic sciences.

Although questions are asked from every subject, they can be categorized as follows: In basic sciences: Anatomy, Biochemistry, Physiology, Histology, Embryology, Microbiology, Pharmacology, and Pathology; and in clinical sciences: Internal Medicine, Pediatrics, General Surgery, Obstetrics, and Gynecology.

The examination covers the entirety of medical education, and everything learned during the six years of medical training can be tested. The MSE consists of two sessions, with the basic sciences test in the morning and the clinical sciences test in the afternoon [8]. The application of the questions to ChatGPT-3.5 and the analysis was conducted to better understand the potential uses of artificial intelligence in medical education and evaluate how artificial intelligence can help medical students.

Selection of questions and application process

The questions used in this study were taken from the 2021 spring term MSE (TUS) booklet, which was last published by ÖSYM [9]. In the evaluation of ChatGPT-3.5, a total of 240 questions in the exam booklet were used. These questions cover both basic medical sciences and clinical medical sciences. In the basic medical sciences section, there were questions from disciplines such as Anatomy, Histology and Embryology, Physiology, Medical Biochemistry, Medical Microbiology, Medical Pathology, and Medical Pharmacology. In the clinical medical sciences section, questions were selected from fields such as Internal Medicine, Pediatrics, Surgery, and Obstetrics.

Each discipline has a certain number of questions and these questions were selected to fully represent the scope of the relevant fields. The questions taken from the 2021 Spring Term MSE (TUS) booklet and the answers to ChatGPT-3.5 were evaluated using the official answer keys published by ÖSYM.

Application of questions to ChatGPT

All questions were submitted in written form in English directly to ChatGPT-3.5. Each question was entered into ChatGPT-3.5 one by one and the model’s answers to these questions were recorded. ChatGPT’s answers to each question were evaluated by comparing them with the official answer keys published by ÖSYM. During this evaluation, the number of correct and incorrect answers ChatGPT gave to each question was recorded.

Analysis

The accuracy of the answers given by ChatGPT-3.5 was evaluated according to the official answer keys of the exam booklet. Each correct answer was considered an indicator of ChatGPT’s performance and the overall success rate was calculated by comparing it with incorrect answers. This analysis was used to reveal ChatGPT’s level of knowledge in the fields of basic and clinical medical sciences and its strengths and weaknesses in these fields.

In this study, 240 questions from the spring session of the 2021 MSE in Turkey were posed to ChatGPT-3.5, and the performance of medical graduates and ChatGPT were compared. The average score for ChatGPT-3.5 was calculated by averaging the minimum and maximum scores. Descriptive statistics are presented. The Mann-Whitney U test was used to compare two independent groups. Statistical calculations were performed using the R.4.0.2 environment. P-values less than 0.05 were considered statistically significant.

## Results

Table 1 presents the number of questions and their respective proportions in the MSE for the subjects of basic medical sciences, i.e., Anatomy, Histology and Embryology, Physiology, Medical Biochemistry, Medical Microbiology, Medical Pathology, and Medical Pharmacology, as well as subjects in clinical medical sciences, i.e., Internal Medicine, Pediatrics, Surgery, and Obstetrics and Gynecology. The lowest weight in the basic medical sciences section was assigned to Histology and Embryology, whereas in the clinical medical sciences section, to Obstetrics and Gynecology [9].

**Table 1:** Information on the name of the test, number of questions, fields, and ratios in the test. *: Ratio in test (%) in the test shows approximately what percentage of the 120 questions in the test area consists of the relevant area.

Test name, number of questions, and duration	Fields	Number of questions in the test	Ratio in test (%)*
Basic medical sciences test, 120 questions, 150 minutes	Anatomy	14	13
	Histology and Embryology	8	7
	Physiology	10	8
	Medical Biochemistry	22	18
	Medical Microbiology	22	18
	Medical Pathology	22	18
	Medical Pharmacology	22	18
Clinical medical sciences test, 120 questions, 150 minutes	Internal Medicine Group	42	35
	Pediatrics	30	25
	Surgery Group	36	30
	Gynecology and Obstetrics	12	10

The candidates’ weighted scores in basic medical sciences and clinical medical sciences were calculated as follows: First, the number of correct and incorrect answers given by candidates in the basic medical sciences test and clinical medical sciences sections test was separately tallied. From the number of correct answers, one-fourth of the number of incorrect answers was subtracted to obtain the raw scores (net numbers) for the basic medical sciences test and clinical medical sciences test. These raw scores were then transformed into standard scores, each with a mean of 50 and a standard deviation of 10, for each test individually. Based on the established standard scores and using the weight coefficients provided in Table 2, the B-score and C-score of each candidate were calculated. For professionals from non-medical fields (biochemistry, pharmacy, chemistry, and veterinary medicine), only the B-score was calculated using the weight coefficients specified in Table 2 [9].

**Table 2:** For medical faculty graduates and other candidates (test weights for candidates attending the medical specialization exam). B: weighted basic medical sciences score; C: weighted clinical medical sciences score; BMST: basic medical sciences test; CMST: clinical medical sciences test

For candidates who graduated from the Faculty of Medicine	BMST standard score	CMST standard score
B score	0.5	0.5
C score	0.7	0.3
For non-medical professional candidates		
C score	1	–

The general score averages for graduates in basic medical sciences ranged from a minimum of 7.51 to a maximum of 81.46 (Table 3). A total of 18,481 people participated in the exam. In clinical medical sciences, the score averages ranged from a minimum of 12.51 to a maximum of 80.78. ChatGPT-3.5, on the other hand, had an average basic medical sciences score with a minimum of 60.00 and a maximum of 72.00. In clinical medical sciences, its average score ranged from a minimum of 66.25 to a maximum of 77.00. For basic medical sciences, the graduates’ average score was 49.68, while ChatGPT-3.5 had an average score of 66.00. In clinical medical sciences, the graduates’ average score was 49.61, and ChatGPT-3.5 had an average score of 71.62. A statistically significant difference was found between the minimum, maximum, and average scores between medical graduates and ChatGPT-3.5 based on basic and clinical medical sciences scores (Table 3) [9].

**Table 3:** Information on B and C scores according to faculty and department information for the 2021 medical specialization exam first term. The minimum score for ChatGPT was calculated assuming all questions were marked and for four incorrect answers, one correct answer was deleted. The maximum score was calculated by taking into account that incorrectly answered questions were not marked. The average score was calculated by taking the average of the minimum and maximum scores. B: weighted basic medical sciences score; C: weighted clinical medical sciences score *: Mann-Whitney U test; p-values <0.05 are statistically significant.

Section/ChatGPT-3.5	B score					C score				
	Number of candidates	Minimum	Maximum	Average	P-value*	Number of candidates	Minimum	Maximum	Average	P-value*
2021-TUS first term										
Medicine	18,457	7.51	81.46	49.68	<0.01	18,457	12.51	80.78	49.61	<0.01
Chat GPT-3.5	1	60.00	72.00	66.00		1	66.25	77.00	71.62	
Biochemistry	4	33.19	40.43	35.13		–	–	–	–	
Pharmacy	2	30.65	41.58	36.12		–	–	–	–	
Chemical	12	26.84	38.91	31.66		–	–	–	–	
Vet	5	36.88	38.91	37.64		–	–	–	–	

While graduates achieved a correct answer rate of 43.3% in basic medical sciences, ChatGPT scored 60.00%. In clinical medical sciences, graduates had a correct answer rate of 53.29%, whereas ChatGPT achieved 63.3%. The field in which ChatGPT excelled the most was Obstetrics and Gynecology, with a correct answer rate of 91.66%, followed by Medical Microbiology with 86.36%. ChatGPT’s least successful area was Anatomy, with a 28.00% correct answer rate in the subfields of basic medical sciences. Graduates outperformed ChatGPT in the Anatomy and Physiology subfields, while ChatGPT exhibited better performance than graduates in all other areas. The least successful areas for graduates were Medical Biochemistry (36.82%), Medical Pharmacology (40.45%), and Medical Microbiology (41.82%). A statistically significant difference was found between the rates of correct answers to the questions on a test basis between medical graduates and ChatGPT-3.5 (p < 0.05) (Table 4).

**Table 4:** ChatGPT-3.5 against the 2021 medical specialization exam first-term test statistics. *: Mann Whitney U test; p-values <0.05 are statistically significant. **: Total number of candidates: 18,481.

Test/Subtest	Graduates**			ChatGPT-3.5			P-value
	Number of questions	True reply number average	Correct answer rate (%)	Number of questions	Number of correct answers	Correct answer rate (%)	
Basic medical sciences test	120	51.63	43.03	120	72	60.00	<0.01
Anatomy	14	6.00	42.86	14	4	28.00	
Histology and Embryology	8	3.59	44.88	8	6	62.50	
Physiology	10	5.94	59.40	10	5	50.00	
Medical Biochemistry	22	8.10	36.82	22	10	45.45	
Medical Microbiology	22	9.20	41.82	22	19	86.36	
Medical Pathology	22	9.90	45.00	22	12	54.54	
Medical Pharmacology	22	8.90	40.45	22	16	72.72	
Clinical medical sciences test	120	63.95	53.29	120	77	63.33	
Internal Medicine Group	42	23.74	56.52	42	25	59.52	
Pediatrics	30	16.29	54.30	30	20	66.66	
Surgery Group	36	18.55	51.53	36	21	58.33	
Obstetrics and Gynecology	12	5.38	44.83	12	11	91.66	

## Discussion

Totlis et al. (2023) [10] found that ChatGPT provides accurate and well-structured anatomical descriptions, including clinical relevance and relationships between structures, concise summaries of sections, and useful advice on anatomical terminology even with complex terms, but not when it comes to anatomical variants and their clinical significance. They reported that the chatbot’s responses were inadequate unless variants were systematically classified by type.

In this study, on a test basis, it was seen that ChatGPT (correct answer rate: 28) showed the worst performance in Anatomy, one of the basic medical sciences subfields, compared to medical graduates (correct answer rate: 42.86). Fields such as Anatomy require visual learning and detailed knowledge. Artificial intelligence models are limited in their ability to interpret and understand visual information because they are trained on text-based data. This can lead to poor performance on visual knowledge-based tests such as Anatomy.

Xu et al. (2024) [11] stated that LLMs offer revolutionary opportunities in medical education, but certain challenges and ethical concerns must be addressed before this technology can be used effectively and safely. Khanam et al (2019) [12] examined the available facts and literature by classifying each type separately and analyzing the scope of their full capabilities to understand the real definitions of artificial intelligence and human intelligence and discussed the possibility of artificial intelligence eventually replacing human work in the market.

In another study conducted by Kung et al. [13] in 2023, ChatGPT’s performance in the United States Medical Licensing Examination (USMLE) Step 1, Step 2 CK, and Step 3 exams was evaluated. This study found that ChatGPT performed near or above the passing threshold on all three exams. ChatGPT demonstrated a high level of consistency and understanding in its explanations, suggesting that LLMs could be helpful in medical education and potentially clinical decision-making.

In a study conducted by Gilson et al. [14] in 2023, the performance of ChatGPT on the USMLE was evaluated. In this study, ChatGPT’s ability to answer questions in USMLE Step 1 and Step 2 exams was discussed and analyzed for user interpretability. ChatGPT was evaluated on two different question sets of 120 free questions provided by AMBOSS and the National Board of Medical Examiners. This evaluation revealed that ChatGPT showed a significant decrease in performance as question difficulty increased. In particular, it was observed that there was a significant decrease in performance as question difficulty increased in the AMBOSS Step 1 dataset.

In our study, ChatGPT (mean B score: 61.98, C score: 61.1) had a higher mean score compared to the graduates (mean B score: 49.68, C score: 49.61) in basic and clinical scores. However, when looking at the maximum scores, medical graduates had a B score of 81.46, while ChatGPT remained at 64.79. Likewise, in the C score, it was seen that medical graduates had a B Score of 80.78, while ChatGPT remained at 63.92. From this, it became clear that graduates, that is, people, will leave artificial intelligence far behind whenever they want.

Subramani et al. (2023) [15] evaluated ChatGPT’s performance in the physiology exam. Responses were evaluated and cross-checked by faculty members with expertise in medical physiology. Overall, ChatGPT passed the Physiology University Exam by a margin (>75% points). In our study, it had 50% correct answer success rate with five incorrect and five correct answers. Liu et al. (2023) [16] discussed the latest advances in ChatGPT research in clinical practice and shared the potential risks and challenges of using ChatGPT in clinical practice. Ashwal et al. (2023) [17] aimed to evaluate the sensitivity, specificity, and accuracy of ChatGPT-3.5, ChatGPT-4, Bing AI, and Bard in predicting drug-drug interactions. In their study, Adeshola and Adepoju (2023) [18] examined its potential impact on student cheating and the challenges and opportunities it presents. By providing insights into ChatGPT, including efforts to address its disruptive nature, it contributes to the existing literature on learning and provides a comprehensive understanding of ChatGPT and its impacts. Lee (2023) [19] conducted research to explore the potential of ChatGPT in medical education amid the rise of this technology. ChatGPT proves itself in various fields. In the study by Eysenbach (2023) [20], an interview with ChatGPT is shared in a study titled “The Role of Productive Language Models and Artificial Intelligence in Medical Education.” Khan et al. (2023) [21] conducted a study on how ChatGPT is reshaping medical education and clinical management. Feng and Shen (2023) [22] worked on how medical educators can harness the power of ChatGPT to create innovative and effective learning experiences for future medical students. Some of the studies in the literature include, in order, the works of Arif et al. (2023) [23], Sallom et al. (2023) [24], Armitage (2023) [25], Abouammoh et al. (2023) [26], and Han et al. (2023) [27]. By offering a comparative analysis between traditional medical education and artificial intelligence-assisted learning and examinations, these studies emphasize the role and effectiveness of technology in education. The findings reveal the extent to which ChatGPT excels in different areas of medical sciences [28-30].

Limitations

This study was conducted using the ChatGPT-3.5 version. Results may vary when new versions or different language models are used. Therefore, it is important to consider that the findings obtained are limited to this specific version and results may vary with advanced models. The study used the number of correct answers as the main criterion in evaluating success. However, other important factors such as students’ clinical skills, practical applications, and knowledge of ethical issues should also be evaluated in medical specialty exams. There has been no evaluation of how ChatGPT performs in such areas of skills and knowledge. The study did not consider the impact of external factors (e.g., exam stress and student motivation) on the results. In real exam conditions, such factors can significantly affect students’ performance. The study evaluated success among ChatGPT and graduates based on only a single exam. A more comprehensive study can be conducted to examine how longer-term learning and performance are affected. The findings are based on a study on a specific exam and a specific artificial intelligence model. Therefore, it may be misleading to assume that the results are directly applicable to other exams or different artificial intelligence models. Additionally, more comprehensive research is needed to evaluate how artificial intelligence models will perform in real medical education settings and across a variety of learning conditions. These limitations should be taken into account when interpreting the results and implications of the study. To develop a more comprehensive understanding of the potential use of artificial intelligence technologies in education, such studies need to be considered in a broader context.

Recommendations

Artificial intelligence models such as ChatGPT can play a significant role in medical education. More medical faculties and educational institutions should provide students with learning opportunities using such technologies. Developing artificial intelligence-supported learning materials and applications for medical education can offer students more resources and test preparation. Artificial intelligence can be used to create personalized educational approaches that better cater to students’ needs. These technologies can be utilized to identify students’ weaknesses and strengthen them. This study points to more research and development opportunities in the field of artificial intelligence and medical education. Studies that delve deeper into the interaction between artificial intelligence and education can help us better understand the potential in this field. Both students and educators in the field of medical education should receive training on the use of artificial intelligence. They should be able to effectively harness this technology. These recommendations can be used to further enhance the role of artificial intelligence in medical education and provide students with a better educational experience.

## Conclusions

This study highlights the potential application of AI, especially LLMs such as ChatGPT, in medical education and specialty exams. The comparative analysis of ChatGPT with medical graduates demonstrates the capabilities and potential of artificial intelligence in this area. More medical schools and educational institutions must adopt and develop artificial intelligence-enhanced learning materials that provide students with additional resources and personalized learning experiences. ChatGPT and similar AI models can help students identify and strengthen their weaknesses, facilitating the development of personalized learning plans. Future developments should focus on further developing and optimizing artificial intelligence-enhanced learning and exam systems, which have the potential to enhance the quality of medical education and improve student outcomes. This study provides insights into future developments in artificial intelligence and education. Artificial intelligence is emerging as an important tool capable of providing significant support to students and making medical education more effective.
